# Enteric neuroanatomy and smooth muscle activity in the western diamondback rattlesnake *(Crotalus atrox)*

**DOI:** 10.1186/s12983-023-00484-1

**Published:** 2023-02-09

**Authors:** Tobias Kohl, Lejla Ridzal, Birgit Kuch, Marlene Hartel, Corinna Kreft, Ahmed Musoski, Klaus Michel, Harald Luksch, Michael Schemann, Anita Annaházi

**Affiliations:** 1 Chair of Zoology, Technical University of Munich, Liesel-Beckmann Str. 4, 85354 Freising, Germany; 2 Chair of Human Biology, Technical University of Munich, Freising, Germany

**Keywords:** Western diamondback rattlesnake, Enteric nervous system, Gastrointestinal motility, Acetylcholine, Nitric oxide, VIP, Tetrodotoxin

## Abstract

**Background:**

Gastrointestinal (GI) functions are controlled by the enteric nervous system (ENS) in vertebrates, but data on snakes are scarce, as most studies were done in mammals. However, the feeding of many snakes, including *Crotalus atrox*, is in strong contrast with mammals, as it consumes an immense, intact prey that is forwarded, stored, and processed by the GI tract. We performed immunohistochemistry in different regions of the GI tract to assess the neuronal density and to quantify cholinergic, nitrergic, and VIPergic enteric neurons. We recorded motility patterns and determined the role of different neurotransmitters in the control of motility. Neuroimaging experiments complemented motility findings.

**Results:**

A well-developed ganglionated myenteric plexus (MP) was found in the oesophagus, stomach, and small and large intestines. In the submucous plexus (SMP) most neurons were scattered individually without forming ganglia. The lowest number of neurons was present in the SMP of the proximal colon, while the highest was in the MP of the oesophagus. The total number of neurons in the ENS was estimated to be approx. 1.5 million. In all regions of the SMP except for the oesophagus more nitric oxide synthase+ than choline-acetyltransferase (ChAT)+ neurons were counted, while in the MP ChAT+ neurons dominated. In the SMP most nerve cells were VIP+, contrary to the MP, where numerous VIP+ nerve fibers but hardly any VIP+ neuronal cell bodies were seen. Regular contractions were observed in muscle strips from the distal stomach, but not from the proximal stomach or the colon. We identified acetylcholine as the main excitatory and nitric oxide as the main inhibitory neurotransmitter. Furthermore, 5-HT and dopamine stimulated, while VIP and the ß-receptor-agonist isoproterenol inhibited motility. ATP had only a minor inhibitory effect. Nerve-evoked contractile responses were sodium-dependent, insensitive to tetrodotoxin (TTX), but sensitive to lidocaine, supported by neuroimaging experiments.

**Conclusions:**

The structure of the ENS, and patterns of gastric and colonic contractile activity of *Crotalus atrox* are strikingly different from mammalian models. However, the main excitatory and inhibitory pathways appear to be conserved. Future studies have to explore how the observed differences are an adaptation to the particular feeding strategy of the snake.

## Background

The gastrointestinal (GI) physiology of snakes is particularly interesting, as many of them feed irregularly with long intervals between meals reaching up to several months or even years [1, 2]. The periods of fasting are compensated by a large amount of food in various foraging snakes, such as rattlesnakes and vipers, where a usual meal consists of ~ 25% of the bodyweight of the animal [3], but it can even be heavier than the snake itself [4]. The storage and processing of such immense whole prey distinguish snakes from most birds and mammals. The function of the gastrointestinal tract in vertebrates is controlled by the enteric nervous system (ENS), which has been described in animals such early in evolution as the cnidarians [5, 6]. However, the vast majority of studies were done in mammals. The ENS has a tremendous capability to adapt. It would therefore be most welcome to know more about comparative neurology and physiology, in particular, to understand the various strategies under evolutionary pressure. In most species, acetylcholine (Ach) was identified as the main neurotransmitter of excitatory and nitric oxide (NO), VIP and ATP that of inhibitory muscle motoneurons [6]. However, although the ENS of snakes must cope with the extraordinary task of storing, digestion, and transit of the massive prey, little is known about the enteric neuroanatomy and neurotransmitters in this species. The rare studies available on the presence of neurotransmitters in the snake GI tract were performed on whole gut extracts [7] or in a semiquantitative manner [1, 8]. Information on the functional role of neurotransmitters in the snake gut is even scarcer. There is an initial report showing that Ach, substance P (SP), bradykinin, and galanin activated the longitudinal muscle layer of the Burmese python stomach and proximal small intestine [1], but the authors did not describe basal motility patterns, nor did they examine the role of NO, which was suggested as an inhibitory neurotransmitter also in the snake, similarly to many other species [8].

Therefore, we aimed to explore basic motility patterns as well as the role of those neurotransmitters, which play a major role in the mammalian gut on gastric, small and large intestinal motility in a venomous foraging snake, the western diamondback rattlesnake (*Crotalus atrox*). Furthermore, we aimed to assess the number of enteric neurons and the ratio of cholinergic, nitrergic, and VIPergic enteric neurons in the different parts of the snake GI tract.

## Results

### Immunohistochemistry

#### Qualitative assessment

**Fig. 1:**
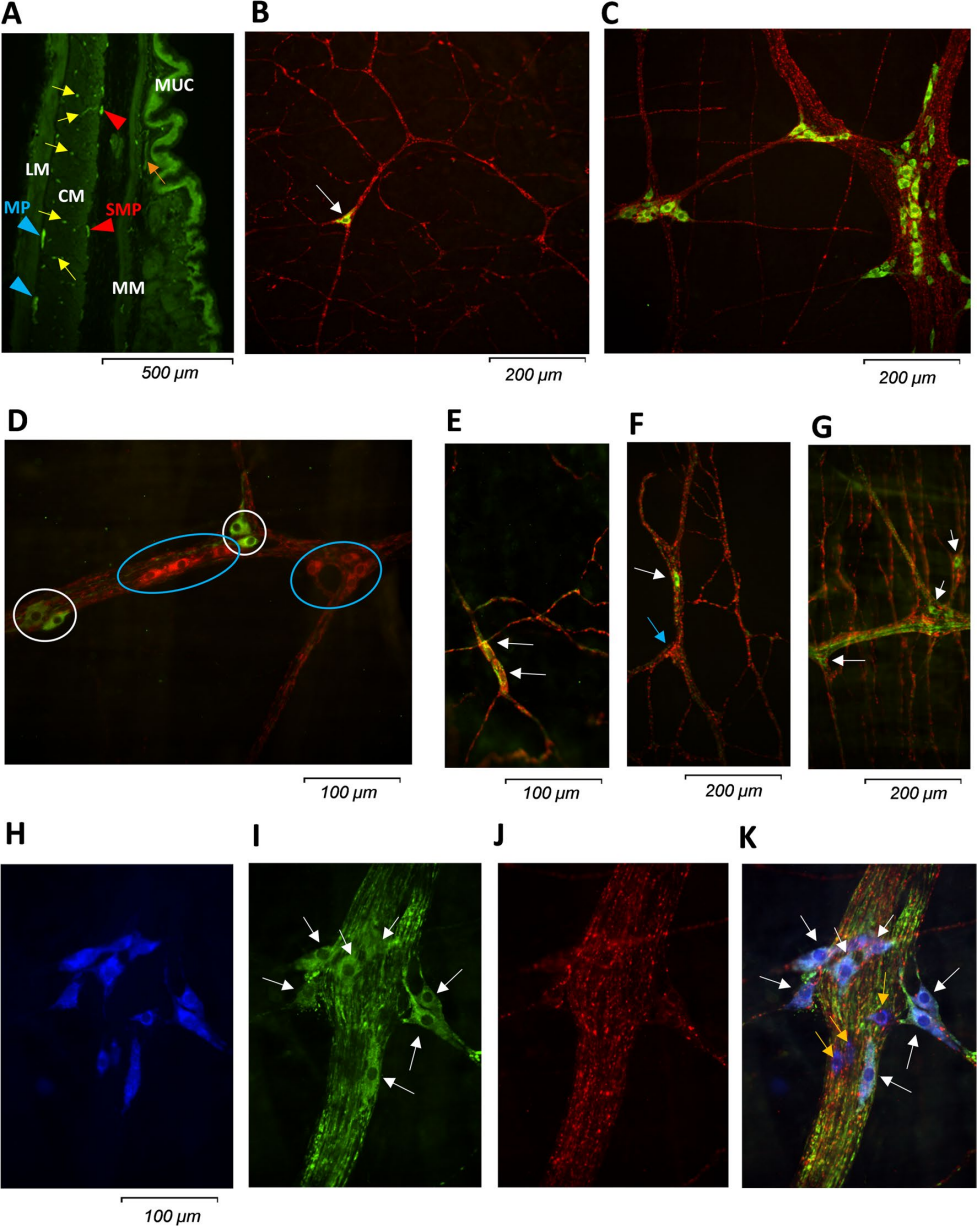
Representative immunofluorescent images of different regions of the GI tract of *Crotalus atrox*. **A** shows a transverse section of the stomach, stained in green for PGP 9.5. Nerve cells of the submucous (SMP) and myenteric plexus (MP) are marked with red and blue arrowheads, respectively. The orange and yellow arrows show neuronal elements innervating the mucosa and the circular muscle layer, respectively. MUC: mucosa, MM: muscularis mucosae, CM: circular muscle, LM: longitudinal muscle. **B**, **C **illustrate the difference between the submucous (SMP; **B**) and myenteric plexus (MP; **C**) of the colon. In the SMP, only one individual neuronal cell body (green, stained for Hu, the pan-neuronal marker; marked with a white arrow) is seen with connecting fiber tracts (red, stained for VIP), while in the MP larger ganglia with several nerve cell bodies (green) are visible. **D** Image of the oesophagus MP demonstrates that ChAT (red) and NOS (green) rarely colocalize and ChAT and NOS positive neurons are generally grouped together (ChAT: blue circle; NOS: white circle). **E**–**G** show examples of VIP-stained nerve cells in different regions and plexuses. In panel **E**, two neuronal cell bodies (white arrows) of the proximal small intestine SMP are visible, which are both stained for NOS (green) and VIP (red). In panel **F**, in the proximal stomach SMP, one neuronal cell body (white arrow) stained for NOS, but not for VIP, and another neuronal cell body (blue arrow) stained for VIP, but not for NOS are present. **G** shows a different colour code in the MP of the distal small intestine. Three neuronal cell bodies are ChAT positive (green, marked with a white arrow), while no cell bodies are stained for VIP (red), only fiber tracts and varicous endings. **H**–**K** presents the same ganglion in the MP of the colon. Panel **H** shows the staining for Hu in blue, which marked all neuronal cell bodies, but no nerve fibers. In panel **I**, several neuronal cell bodies (marked with white arrows) are stained for TH (green), corresponding to dopaminergic neurons. In panel **J**, no cell bodies, only fibers are stained with DBH (red), a marker of noradrenalin synthesis. **K** shows the merged image of all 3 stainings. Note that apart from blue, most neuronal cell bodies are also stained for TH with green (white arrows), but three neuronal cell bodies (orange arrows) appear in pure blue colour only, representing TH-negative nerve cells

In the transverse sections, two plexuses of the ENS could be identified in all examined regions: one submucous plexus (SMP) between the mucosa and the circular muscle and a myenteric plexus (MP) between the circular and longitudinal muscle (Fig. 1A).

In the oesophagus, the SMP consisted of small ganglia containing a few cell bodies and scattered single neuronal cell bodies outside the ganglia. In the myenteric plexus, the ganglia were larger with more cell bodies than in the SMP. In the stomach, similar to the oesophagus, a few cell bodies forming small ganglia were observed in the SMP, but mostly the submucous layer consisted of single neuronal cell bodies. In the distal stomach, the neuronal network was much denser compared to the proximal stomach. In the MP, both in the proximal and the distal part, larger and more numerous ganglia were seen with multiple neurons, compared to the SMP. In the small intestine SMP, many nerve fibers were observed running along the blood vessels and from proximal to distal along the folds of the mucosa, but only a few, individual neurons were found. The MP showed a dense, net-like structure with large ganglia. The SMP in the colon consisted of sparse fiber tracts connecting single neuronal cells (Fig. 1B). The MP was similar to that of the small intestine, with a dense network of interganglionic fiber tracts, mostly running parallel to the circular muscle, with different sized ganglia (Fig. 1C). In the proximal colon, the MP appeared denser in the stretched tissue than in the distal colon.

#### Quantitative assessment

For each anatomical region, the stretch factor was calculated individually for each of the three snakes (Table 1). The oesophagus was the most elastic, followed by the distal colon and the proximal small bowel. The distal small bowel, proximal stomach, and proximal colon were less elastic. The least elastic part was the distal stomach.

**Table 1:** Total length and diameter, stretch factor, neuronal density, and the total number of neurons of the different gastrointestinal regions

	Total length (l total, cm)	Diameter (d unstretched, cm)	Stretch factor	Neuronal density (1/cm^2^)		Total number of neurons	
				MP	SMP	MP	SMP
Oesophagus	17.3	0.9	13.7	60.578 ± 16.575*	6.067 ± 4.687*	908.690 ± 186.780	91.494 ± 71.922
Prox. stomach	1.5	2.0	4.3	29.480 ± 13.967	17.335 ± 10.453	85.057 ± 35.620	49.857 ± 29.610
Distal stomach	1.5	1.8	1.8	15.133 ± 1.880	7.777 ± 7.338	39.576 ± 4.682*	21.058 ± 21.431
Prox. small bowel	5.5	0.9	5.3	25.128 ± 3.475	394 ± 258^§^	119.184 ± 32.058^§^	2.613 ± 1.696
Distal small bowel	3.5	0.8	5.0	40.116 ± 11.999	623 ± 350^§^	102.846 ± 31.088^§^	1.590 ± 975*^§^
Prox. colon	1.7	0.7	3.0	43.849 ± 13.073	690 ± 325^§^	51.395 ± 10.174*	818 ± 435*^§^
Distal colon	2.4	1.0	5.6	27.020 ± 12.386	1.071 ± 358	65.300 ± 24.903*	2.689 ± 948
				*Significantly different from distal stomach	*Significantly different from proximal small bowel ^§^significantly different from proximal stomach	*Significantly different from oesophagus ^§^significantly different from distal stomach	*Significantly different from oesophagus ^§^significantly different from proximal stomach

In the table, average results are shown, while during extrapolating the neuronal density to the total number of neurons for each animal, the total surface area was calculated for the individual animals and therefore deviates from the product of length and circumference

The calculated neuronal densities for each region can be seen in Table 1. The highest neuronal density was in the MP of the oesophagus, while the lowest was in the SMP of the proximal small bowel. In all regions, the neuronal density was higher in the MP than in the SMP.

The total number of neurons was calculated based on the unstretched total surface of each region, multiplied by the neuronal density (Table 1). The highest number of neurons was found in the MP of the oesophagus, and the smallest in the SMP of the proximal colon.

**Fig. 2:**
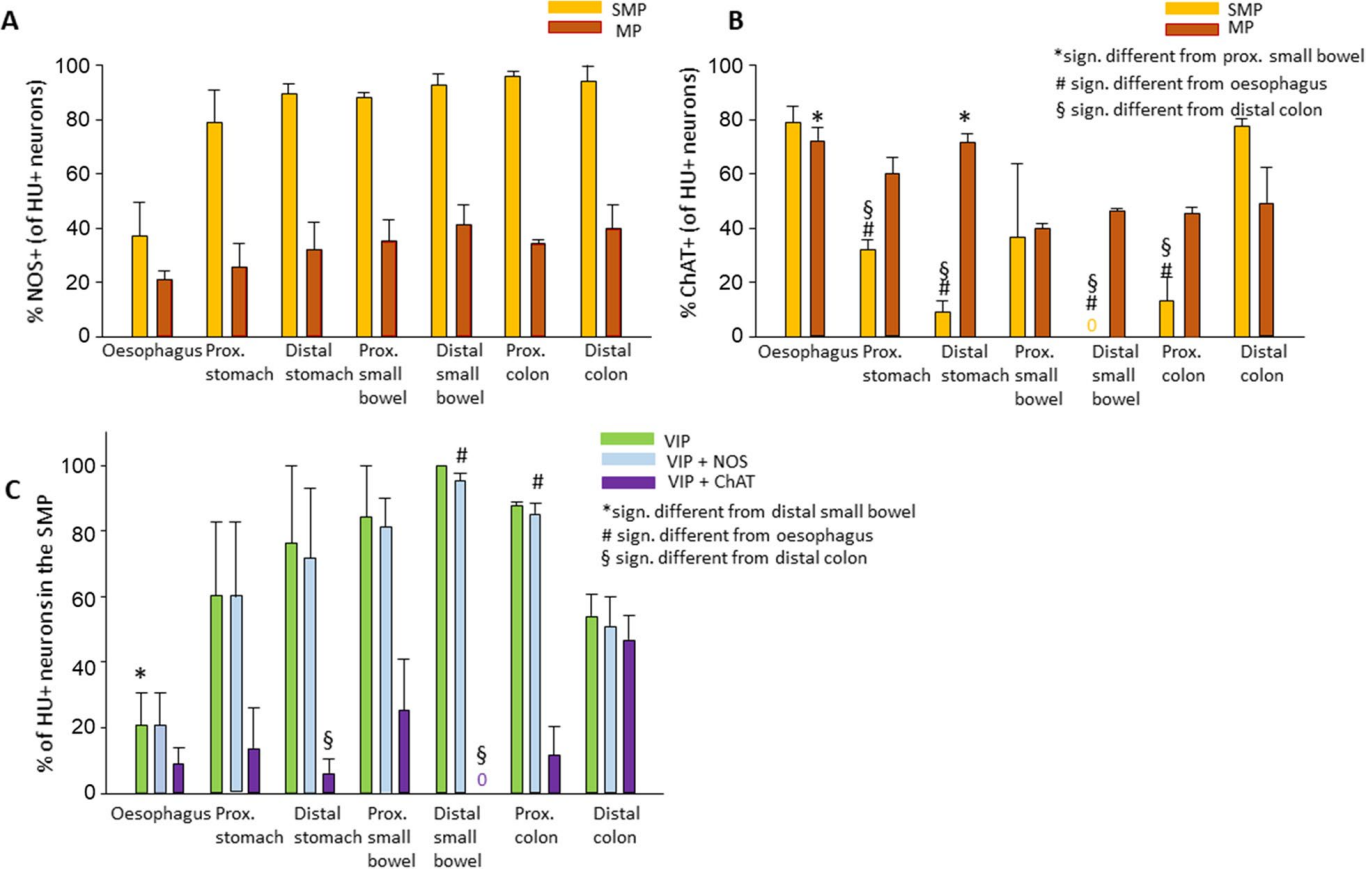
**A**, **B** Percentage of NOS (**A**) and ChAT (**B**) positive neurons (of all cells stained for the neuronal marker, HU) in the submucous and myenteric plexus of the different regions of the GI tract. **C** Percentage of neurons (of all cells stained for the neuronal marker, HU) positive for VIP, VIP and NOS and VIP+ ChAT in the submucous plexus

In all regions of the MP, NOS+ and ChAT+ neurons were present (Fig. 2A, B). In the SMP of the distal stomach, distal small intestine, and proximal colon, mostly NOS+, and few or no ChAT+ neurons were seen.

We calculated the percentage of ChAT+, and NOS+ neurons in both plexuses of each region (Fig. 2A, B). In all regions, the MP contained more ChAT+ than NOS+ neurons, while in the SMP, except for the oesophagus, most of the neurons were NOS+. There was no significant difference between the percentage of NOS+ neurons in either the submucous or in the myenteric plexus (Fig. 2A). However, in the submucous plexus (Fig. 2B), ChAT+ neurons were more numerous in the oesophagus (78.9 ± 6.1%) and the distal colon (77.5 ± 2.5%) than in the proximal (32.1 ± 3.8%; *p* < 0.001) and distal stomach (8.9 ± 4.5%, *p* < 0.001), distal small bowel (0 ± 0%, *p* < 0.001) and proximal colon (13.4 ± 8.3%, *p* < 0.001). In the myenteric plexus, a significantly higher percentage of neurons were ChAT+ in the oesophagus (72.3 ± 4.8%, *p* = 0.021) and distal stomach (71.1 ± 3.1%, *p* = 0.025), than in the proximal small bowel (39.8 ± 2.1%).

There were almost no VIP+ cell bodies in the MP (2 cell bodies in all 84 areas, Fig. 1G). However, in the SMP, most cell bodies were stained for VIP (Fig. 1E, F). The percentage of VIP-stained neurons was significantly higher in the distal small intestine (100 ± 0%, *p* = 0.02) than in the oesophagus (20.7 ± 9.9%, Fig. 2C). When analyzing the co-staining of VIP with NOS, most neurons were stained for both in all examined regions (Fig. 1E). However, co-stainings with ChAT were very rare, except for the distal colon. A significantly higher percentage of neurons were positive for both VIP and NOS in the distal small bowel (95.2 ± 2.4%, *p* = 0.019) and the proximal colon (85.3 ± 3.3%, *p* = 0.049) than in the oesophagus (20.7 ± 9.9%). Co-staining with ChAT appeared more frequently in the distal colon (46.8 ± 7.4%) than in the distal small intestine (0 ± 0%, *p* = 0.013) and in the distal stomach (6.2 ± 4.2%, *p* = 0.035).

ChAT, NOS, and VIP positive fiber tracts were all visible in the muscle layer in the MP preparations, confirming that they belong to motoneurons innervating the muscle.

Staining for TH and DBH was performed only in one snake in the stomach and the colon myenteric plexus, therefore the results were not quantified (Fig. 1H–K). Numerous cell bodies were observed with TH staining, but no DBH staining, corresponding to dopaminergic neurons (Fig. 1I). DBH staining was extensive but only present in nerve fibers, not in neuronal cell bodies (Fig. 1J).

### Motility experiments

The baseline activity patterns showed region-specific features. In the distal, but not in the proximal stomach phasic, small amplitude contractions with 0.3–0.9/min frequency prevailed. The proximal stomach showed no regular changes in the tone, sometimes spontaneous, irregular contractions occurred with an overall frequency between 0–7/hour and force up to 5 mN. The colon exhibited the most stable muscle tone compared to the proximal and distal stomach, spontaneous contractions were rarely present.

The three different regions, proximal and distal stomach and colon showed distinct responses to electrical field stimulation (EFS). The contractile on-responses in the proximal (n = 18; 9.3 [2.7/18.7] mN) and distal (n = 36; 14.2 [8.6/28.2] mN) stomach were significantly larger than those in the colon (n = 31; 1.0 [0.1/1.8]; both *p* < 0.001). There was no significant difference between the regions in the magnitude of the late response, which consisted of a small decrease in muscle tone in the majority of cases (prox. stomach: − 1.6 [− 4.2/− 0.2] mN; distal stomach: − 0.8 [− 1.7/− 0.5] mN; colon: − 2.0 [− 3.1/− 1.1]; *p* = 0.326). However, deviations from this pattern have been often observed, particularly in the proximal stomach. Here, in 47% of cases, the late response was missing, and in 18% it was a re-contraction instead of a relaxation. In the distal stomach, the late response was absent in 8% and a contraction appeared in only 0.8%. In the colon, no late response was seen in 28% and contraction in 2% of cases. Only in the colon, the contractile on-response was absent in 16% of cases, but a contractile late response was present. As the late response was small and unreliable, it was not further analyzed in the pharmacological experiments.

The numerical results of the pharmacological experiments are shown in Table 2 for the proximal stomach, Table 3 for the distal stomach, and Table 4 for the colon.

**Table 2:** The effect of different drugs on the motility of the proximal stomach

Drug	N (tissue/animal)	Tone Before (mN)	Tone After (mN)	*p* value	EFS contractile on before (mN)	EFS contractile on after (mN)	*p* value
Ach	9/5	8.9 [6.8/16.1]	11.8 [9.1/27.3]	**0.047**			
Atropine	11/6	10.4 [8.1/20.9]	9.4 [6.3/20.2]	0.52	7.4 [1.9/18.0]	0.6 [-0.1/5.4]	**0.039**
L-NAME	6/5	6.3 [5.0/7.4]	7.4 [6.5/19.5]	**0.031**	9.1 [1.6/15.5]	14.4 [3.2/25.2]	**0.007**
SNP	5/3	24.3 [14.5/41.8]	3.6 [2.1/6.1]	**0.021**			
5-HT	8/4	5.4 [3.6/8.2]	14.5 [11.0/15.8]	**0.006**			
Dopamine	7/5	6.0 [4.7/9.1]	4.7 [3.6/9.6]	0.375			
Domperidone	7/4	15.3 [10.7/22.3]	11.9 [9.1/17.3]	0.156	10.6 [6.2/16.3]	12.1 [2.8/16.3]	0.668
VIP	7/5	16.0 [6.3/17.7]	6.9 [4.0/15.0]	**0.014**			
Suramin	9/5	7.6 [7.4/17.6]	7.3 [3.0/18.1]	0.461	16.7 [8.4/22.1]	17.7 [8.7/27.5]	0.148
Propranolol	8/5	6.3 [3.3/7.3]	7.9 [3.9/16.8]	0.148	8.9 [4.6/19.3]	23.3 [17.5/39.2]	**0.004**
Isoproterenol	8/6	9.8 [5.5/12.6]	3.3 [1.8/6.9]	**0.008**	13.0 [6.7/17.7]	2.6 [1.6/4.9]	**0.012**
TTX	6/4	5.6 [5.0/22.6]	5.9 [4.4/18.7]	0.313	13.2 [9.1/21.6]	12.7 [7.1/20.1]	0.563
Lidocaine	8/6	6.8 [5.1/12.9]	21.6 [19.8/31.9]	**< 0.001**	9.3 [6.1/17.6]	0.0 [-0.7/0.4]	**0.008**
Na-free Krebs sol	7/4	12.5 [10.4/15.0]	74.9 [70.9/90.6]	**0.016**	16.4 [4.3/19.5]	0.0 [0.0/0.0]	**0.016**

Changes in the muscle tone and of the response to EFS after the application of different drugs. Bold letters mean a significant effect

**Table 3:** The effect of different drugs on the motility of the distal stomach

Drug	N (tissue/animal)	Tone Before (mN)	Tone After (mN)	*p* value	Ampl. Before (mN)	Ampl. After (mN)	*p* value	Freq. Before (1/min)	Freq. After (1/min)	*p* value	EFS contractile on before (mN)	EFS contractile on after (mN)	*p* value
Ach	11/7	7.8 [5.9/11.8]	8.8 [7.4/19.6]	**0.002**	1.6 [0.6/7.6]	14.1 [6.9/31.7]	**0.031**	0.4 ± 0.1	0.7 ± 0.1	**0.01**			
Atropine	14/11	9.4 [7.2/10.8]	9.1 [6.4/10.1]	**0.005**	5.3 [3.0/12.2]	3.3 [2.0/9.6]	**< 0.001**	0.7 ± 0.1	0.6 ± 0.1	0.739	16.0 [6.2/34.9]	1.8 [0.0/4.7]	**< 0.001**
L-NAME	16/9	10.6 [4.7/14.6]	10.5 [6.6/13.4]	0.98	4.1 [2.0/10.6]	22.5 [7.9/35.5]	**< 0.001**	0.5 ± 0.3	0.6 ± 0.2	0.389	21.4 [8.6/37.4)	98.1 [62.9/119.2]	**< 0.001**
SNP	8/6	7.6 [5.5/12.0]	6.1 [3.6/8.6]	**0.008**	12.4 [5.5/15.2]	0.5 [0/1.7]	**0.008**	0.9 ± 0.1	0.5 ± 0.2	0.101			
5-HT	7/4	6.2 [4.4/8.6]	7.1 [6.2/8.9]	**0.016**	1 [0/1.4]	3.5 [2.2/14.4]	**0.016**	0.6 ± 0.5	1 ± 0.2	0.0775			
Dopamine	8/4	6.0 [3.7/8.3]	8.0 [4.7/9.7]	**0.039**	5.1 [0.4/6.6]	10.2 [1.8/13.4]	**0.042**	0.7 ± 0.3	0.9 ± 0.4	**0.001**			
Domperidone	7/3	10.0 [8.8/14.1]	10.4 [7.9/14.0]	1	6.0 [4.4/8.0]	7.9 [4.8/12.0]	0.563	0.7 ± 0.2	0.7 ± 0.1	0.734	74.0 [33.6/92.9]	31.4 [19.0/81.7]	0.375
VIP	7/4	8.6 [7.4/11.0]	7.4 [6.3/9.8]	**0.031**	2.2 [0.3/7.9]	0.8 [0.4/2.2]	0.219	0.6 ± 0.1	0.5 ± 0.1	0.368			
Suramin	9/4	12.8 [5.5/15.0]	12.6 [5.4/14.0]	0.359	8.0 [4.2/13.6]	11.9 [7.1/16.5]	**0.031**	0.7 ± 0.1	0.7 ± 0.1	0.341	22.8 [0.1/27.7]	16.3 [2.9/28.6]	0.938
Propranolol	9/8	5.0 [3.6/10.3]	5.6 [3.9/10.2]	0.359	5.5 [4.1/6.7]	6.0 [3.4/8.7]	0.359	0.7 ± 0.1	0.7 ± 0.1	0.526	35.2 [15.2/57.2]	45.1 [19.9/62.1	**0.031**
Isoproterenol	11/7	7.2 [6.2/10.4]	4.5 [3.3/6.4]	**< 0.001**	4.6 [2.9/9.1]	0.0 [0.0/2.1]	**0.008**	0.7 ± 0.1	0.4 ± 0.2	**0.025**	26.5 [8.8/74.0]	4.80 [2.2/10.0]	**0.031**
TTX	7/4	8.5 [8.3/13.1]	8.7 [8.1/12.2]	0.219	13.8 [3.1/30.5]	15.8 [10.2/26.7]	0.844	0.3 ± 0.1	0.4 ± 0.1	0.332	17.1 [8.6/64.4]	42.5 [3.0/55.3]	0.938
Lidocaine	8/6	10.8 [4.8/14]	18.9 [1.7/31.5]	**0.021**	4.4 [3.6/6.9)	5.9 [4.1/13.0]	0.742	0.6 ± 0.1	0.8 ± 0.1	**0.012**	1.9 [1.2/32.5]	0.0 [0.0/0.3]	**0.004**
Na-free Krebs sol	6/3	7.5 [3.9/12.0]	25.6 [19.3/33.7]	**0.031**	2.4 [1.0/3.9]	19.6 [6.9/25.8]	**0.031**	0.7 ± 0.2	1.1 ± 0.01	**0.029**	7.6 [4.3/10.6]	0.0 [0.0/0.6]	**0.031**

Changes of the muscle tone, of the frequency and amplitude of spontaneous contractions, and of the response to EFS after the application of different drugs. Bold letters mean a significant effect

**Table 4:** The effect of different drugs on the motility of the colon

Drug	N (tissue/animal)	Tone before (mN)	Tone after (mN)	*p* value	EFS contractile on before (mN)	EFS contractile on after (mN)	*p* value
Ach	12/8	8.3 [5.2/11.8]	9.2 [7.5/12.8]	**0.002**			
Atropine	12/10	12.3 [4.6/18.6]	11.7 [4.1/19.2]	0.42	2.1 [1.0/8.8]	0.8 [0.1/2.1]	**0.008**
L-NAME	10/7	11.9 [9.7/22.5]	12.7 [11.0/21.9]	**0.049**	0.9 [0.3/2.6]	3.6 [2.4/7.3]	**0.004**
SNP	11/7	17.2 [5.8/19.9]	4.9 [3.7/14.3]	**< 0.001**			
5-HT	7/4	5.9 [3.8/8.2]	4.3 [3.5/5.4]	0.109			
Dopamine	7/4	11.5 [7.4/12.4]	10.8 [6.9/12.0]	0.69			
Domperidone	6/3	12.1 [10.6/13.2]	12.4 [10.6/13.6]	0.438	1.5 [1.1/4.2]	2.1 [1.6/3.9]	0.438
VIP	7/4	10.3 [8.4/15.6]	8.3 [7.1/14.5]	**0.036**			
Suramin	10/5	10.3 [5.9/13.5]	9.7 [4.8/12.6]	0.084	0.4 [0.1/1.2]	0.4 [0.1/1.7]	0.461
Propranolol	8/6	9.1 [7.4/10.3]	8.9 [7.3/10.4]	0.938	0.1 [0.0/1.2]	0.1 [0.0/1.5]	0.5
Isoproterenol	11/7	10.9 [8.8/14.3]	8.7 [6.0/10.0]	**0.002**	1.7 [0.9/4.4]	0.3 [0.2/2.0]	**0.027**
TTX	11/6	6.2 [4.0/13.1]	6.3 [4.5/12.8]	0.898	1.4 [0.4/5.0]	1.9 [0.8/3.6]	1
Lidocaine	14/7	6.5 [3.9/10.3]	17.5 [7.6/21.1]	**< 0.001**	2.6 [1.3/5.7]	0.0 [0.0/0.8]	**0.002**
Na-free Krebs sol	7/4	9.6 [6.7/10.4]	21.0 [16.8/23.0]	**0.016**	1.8 [0.5/3.8]	0.0 [0.0/0.3]	**0.008**

Changes in the muscle tone and of the response to EFS after the application of different drugs. Bold letters mean a significant effect

**Fig. 3:**
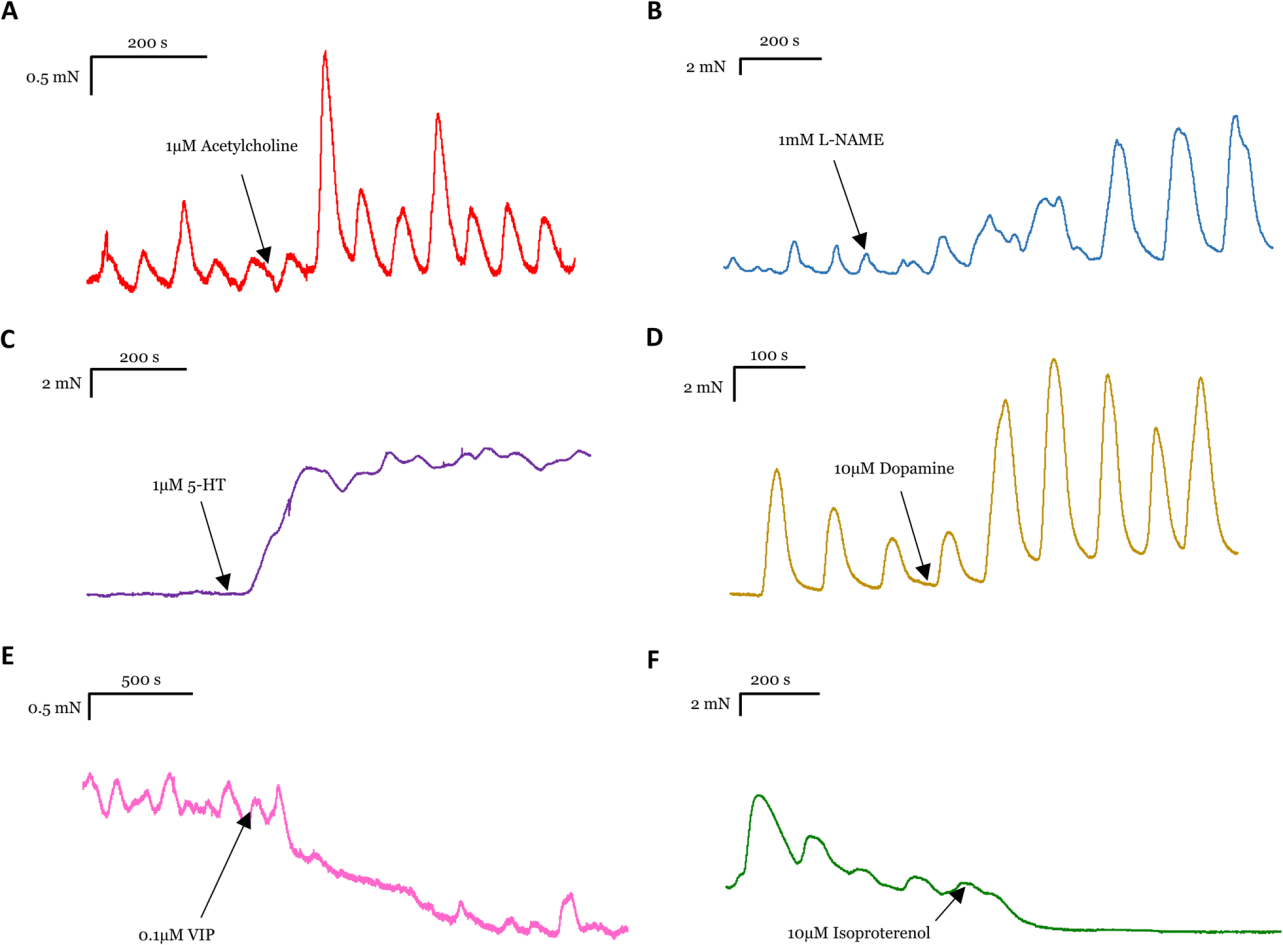
Original traces of organ bath experiments showing examples of the effect of different drugs in different GI regions. **A** Ach increased the tone, and the amplitude and frequency of spontaneous contractions in the distal stomach; **B** L-NAME increased the tone in the colon; **C** serotonin increased the tone in the proximal stomach; **D** dopamine increased the tone, the amplitude, and frequency of spontaneous contractions in the distal stomach; **E** VIP decreased the tone in the colon; **F** isoproterenol reduced the tone in the proximal stomach

Ach increased muscle tone in all examined regions significantly (Fig. 3A). In the proximal stomach, the tone was increased by 33%, in the distal stomach by 13%, while in the colon by 11%. Ach also increased the amplitude by a factor of 9 and the frequency by 75% of that observed during the pre-Ach period in the distal stomach significantly.

Atropine did not change the basal tone in the proximal stomach or the colon. However, it caused a small but significant decrease in the tone in the distal stomach by 3%. Furthermore, it dramatically reduced the contractile on-response in all three regions. In the proximal stomach, the response was reduced to 8%, in the distal stomach to 11%, and in the colon to 38%. Atropine significantly decreased the amplitude of spontaneous contractions in the distal stomach by 38% but did not influence their frequency.

L-NAME (Fig. 3B) significantly increased the basal tone in the proximal stomach by 17% and in the colon by 7% but it did not change the muscle tone in the distal stomach. L-NAME increased the contractile on-response significantly in the proximal stomach by 58%, in the distal stomach by 358%, and in the colon by 300%. The frequency of spontaneous contractions in the distal stomach was unchanged by L-NAME. The amplitude of spontaneous contractions was significantly increased by 449%.

SNP significantly decreased the muscle tone in all regions, in the proximal stomach by 85%, in the distal stomach by 20%, and in the colon by 72%. SNP also dramatically reduced the amplitude of spontaneous contractions in the distal stomach by 96% but did not significantly alter the frequency.

Serotonin (5-HT) significantly increased the tone in the proximal stomach by 169% and in the distal stomach by 15%, but had no effect in the colon (Fig. 3C). The amplitude of the spontaneous contractions in the distal stomach were significantly increased by 250%.

Dopamine caused a significant increase in the basal tone by 33% only in the distal stomach, but not in the proximal stomach or colon (Fig. 3D). In the distal stomach, it significantly increased the amplitude and the frequency of spontaneous contractions by 100% and by 34%, respectively.

Domperidone did not change the muscle tone in any of the regions. It also did not alter the on- response in any of the regions. Domperidone did not change the amplitude and frequency of spontaneous contractions in the distal stomach.

VIP caused a significant relaxation in all regions, in the proximal stomach by 57%, in the distal stomach by 8%, and in the colon by 19% (Fig. 3E). VIP did not change the amplitude and frequency of spontaneous contractions in the distal stomach.

Suramin did not change the basal tone in any of the regions. Suramin had no effect on the contractile on-response in any of the regions. Suramin did not change the frequency of spontaneous contractions in the distal stomach, but it increased the amplitude by 49%.

Propranolol did not change the muscle tone in any of the regions. Propranolol significantly increased the contractile on-response in the proximal stomach by 162% and in the distal stomach by 28%, but not in the colon. The frequency and amplitude of spontaneous contractions in the distal stomach were unchanged by propranolol.

Isoproterenol reduced the muscle tone in all regions significantly (Fig. 3F). The change was -66%, -37%, and -20% in the proximal stomach, distal stomach, and colon, respectively. It also reduced the on-response in all regions significantly, in the proximal stomach by 80%, in the distal stomach by 82%, and in the colon also by 82%. Isoproterenol also significantly reduced the frequency of the spontaneous contractions in the distal stomach to the half and the amplitude was almost reduced to zero.

**Fig. 4:**
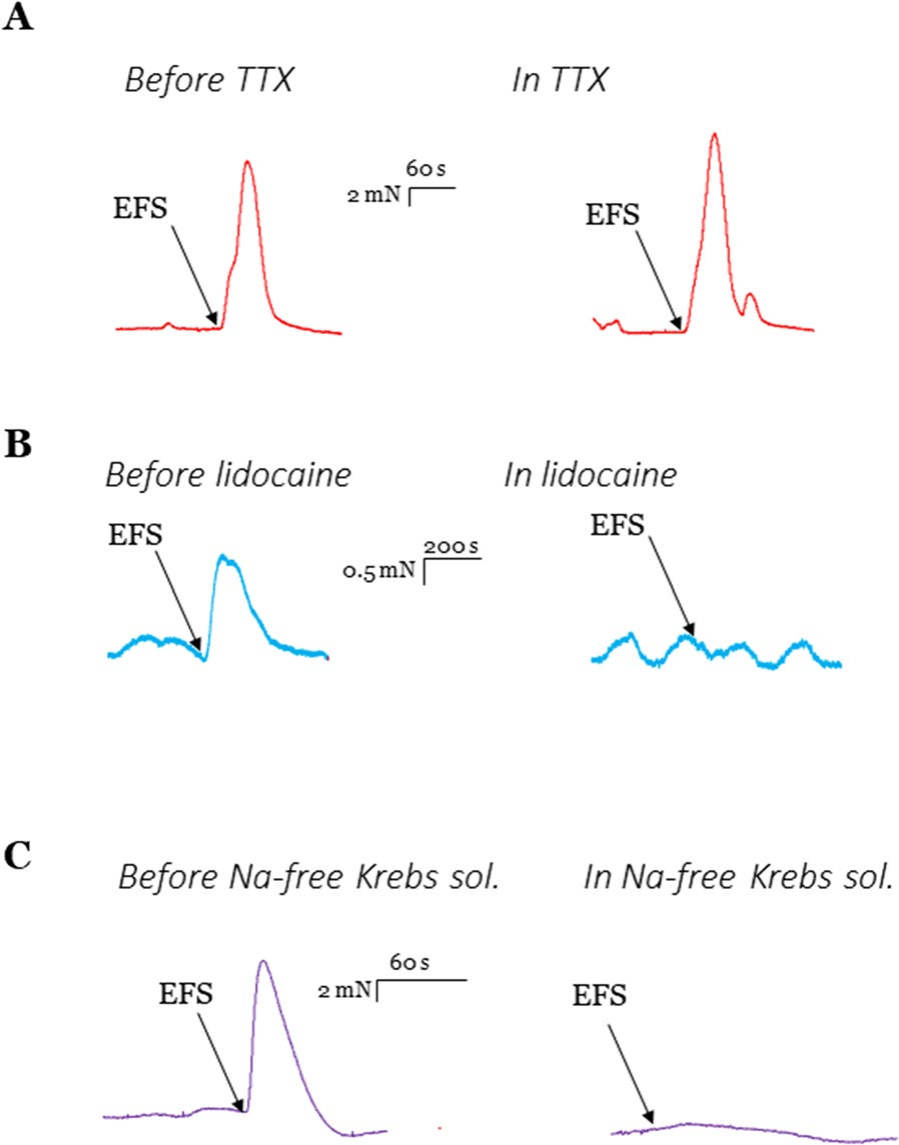
Original traces of organ bath experiments showing examples of the effect of TTX, lidocaine, and sodium-free Krebs-solution on the response to EFS in different GI regions. **A** TTX did not change the on-response to EFS in the distal stomach. **B** Lidocaine abolished the on-response to EFS in the colon. **C** Sodium-free Krebs-solution abolished the on-response to EFS in the proximal stomach

TTX did not change the basal tone in any of the tested regions. TTX also had no influence on the contractile on-response (Fig. 4A). TTX had no effect on the amplitude and frequency of the spontaneous contractions in the distal stomach.

Lidocaine significantly increased the baseline tone in all regions. In the proximal stomach, the tone was increased by 218%, in the distal stomach by 75%, and in the colon by 169%. Lidocaine completely abolished the contractile on-response in all regions (Fig. 4B). Lidocaine did not change the amplitude of spontaneous contractions in the distal stomach, but it increased their frequency by 33%.

Na-free Krebs solution significantly increased the muscle tone in all regions. Namely, in the proximal stomach, the tone was increased by 500%, in the distal stomach by 241%, and in the colon by 119%. Na-free Krebs solution completely abolished the on-response in all regions (Fig. 4C). In the distal stomach Na-free Krebs solution significantly increased the frequency of spontaneous contractions by 57% and the amplitude by 717%.

### Neuroimaging experiments

**Fig. 5:**
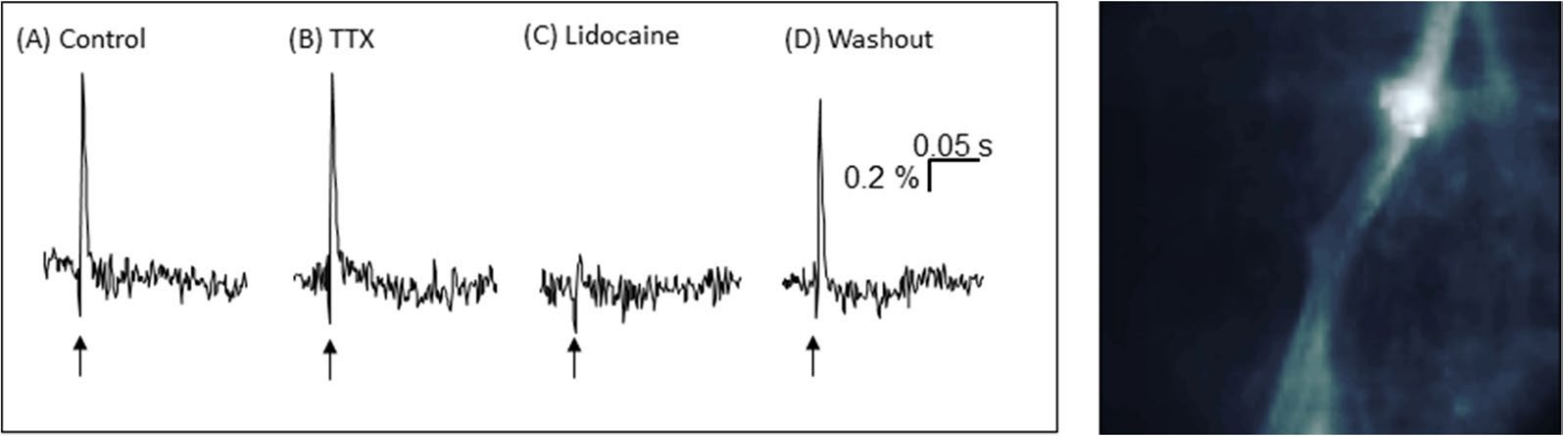
*Left:* Snake proximal colon myenteric plexus ganglia are insensitive to TTX, but sensitive to lidocaine. Arrows correspond to EFS. **A** Control response to a single pulse electrical field stimulation (EFS) of an interganglionic fiber tract. EFS after **B** 15 min perfusion of 1 µM TTX, **C** 15 min perfusion of 1 mM lidocaine, **D** 1 h washout period following lidocaine perfusion. *Right:* Snake myenteric plexus stained with di-8-ANEPPS with electrode placement near the top of the fiber tract

Electrophysiological experiments in the colonic myenteric plexus were performed to support TTX-insensitive spikes. (Fig. 5) The experiments were performed on two tissues from two individual animals (N = 2). A single electrical stimulus to interganglionic fiber tracts evoked compound action potentials, generated by numerous (probably thousands) passing nerve fibers [9]. Those compound action potentials were insensitive to 1 µM TTX but blocked by 1 mM lidocaine. The compound action potentials returned after a 1 h washout period.

## Discussion

With our study, we provided for the first time data on the number of nerve cells in the ENS of a snake (*Crotalus atrox)*. Moreover, we assessed the basic neurochemical code of enteric neurons by counting cholinergic and nitrergic neurons in both the submucous and myenteric plexus of the esophagus, stomach, and small and large intestine. We also demonstrated the presence of VIP-containing neurons, mainly in the submucous plexus in all examined regions, and dopaminergic neurons in the myenteric plexus of the stomach and colon. Furthermore, after describing basic motility patterns in the organ bath, we have confirmed the role of ACh and NO as the primary neurotransmitters of excitatory and inhibitory muscle motoneurons, respectively. Additionally, we demonstrated that 5-HT and dopamine exerted an excitatory effect, while VIP and the ß-receptor-agonist isoproterenol showed an inhibitory effect on the motility. ATP has probably only a marginal effect, which is inhibitory. Finally, we have shown that voltage-gated Na-channels on enteric neurons of *Crotalus atrox* are TTX insensitive, but sensitive to lidocaine.

We have found a well-developed myenteric plexus with ganglia consisting of several neurons in all of the studied regions in *Crotalus atrox*. This finding was similar to mammals like the guinea pig or humans, contrary to fish which lack well-organized plexuses and whose neurons are rather scattered individually or form small groups at the nodes of fiber connections [10]. The density of neurons in the myenteric plexus did not significantly differ between the parts of the gut, except for the oesophagus, where neurons were significantly denser compared to the distal stomach. However, the submucous plexus was not well-developed based on the finding that a substantial proportion of enteric neurons were scattered rather than organized in ganglia. In addition, submucous nerve cell bodies were even scarcer in the intestines, confirmed by a significantly lower neuronal density compared to the proximal gut regions, although nerve fibers could be readily observed. This is in accordance with previous observations in the Burmese python, where nerve cell bodies were almost completely limited to the myenteric plexus in the small and large intestines (the esophagus and stomach were not examined) [1]. Similarly, in the common garter snake, *Thamnophis sirtalis*, no submucous plexus could be observed in the small and large intestines [8].

Data on neuronal densities and the total number of neurons in the ENS in all species are generally scarce and completely lacking for snakes. Most studies provide quantitative data only for a single gut region and the variations in the methodology limit the comparability between data from different sources. However, our group has recently completed a work assessing the neuronal density and the total number of neurons in three mammals: mouse, guinea pig, and human [11]. In this study, our group has calculated the total number of neurons in the ENS to be 2.6 million for mice, 14.6 million for guinea pigs, and 168 million for humans. Our result of 1.5 million in the rattlesnake may suggest that reptiles have much fewer enteric neurons than mice, despite similar body weights (32.2 ± 7.5 g for snakes used for the neuronal count *vs*. 24.5 ± 3.4 g in case of mice). In case of the myenteric plexus, the neuronal density varied from 15,133 ± 1,880 to 60,578 ± 16,575 1/cm^2^ in the different regions of the snake GI tract, which was comparable to the averages found in the mouse (35,011 ± 25,017 1/cm^2^), guinea pig (24,315 ± 16,627 1/cm^2^) and human (21,698 ± 9,492 1/cm^2^). Among these three mammals, the highest neuronal density was observed in the mouse duodenum (45,771 1/cm^2^), which is still under the value of neuronal density in the snake oesophagus (60,578 ± 16,575 1/cm^2^). Even more striking was the finding, that while the highest neuronal density of the snake was observed in the myenteric plexus of the oesophagus, this region had the lowest neuronal density in the mouse and guinea pig. We may speculate that it is related to the distinct function of the oesophagus in the snake, which has to accommodate and push the large undigested whole prey in direction of the stomach. A further important difference is the low neuronal densities in all regions of the submucous plexus of the snake, which becomes even more evident in the small and large intestines, as opposed to the three mammals, which possess a well-developed submucous network in these regions.

Our study is also unique in that it provided a quantitative analysis of cholinergic and nitrergic neurons in the ENS of snakes. In a previous study, several hormones and neuropeptides have been assessed in the plasma and gastrointestinal tissues of Burmese pythons, including CGRP, VIP, and SP [7]. However, as the substances were measured in whole gut extracts, no differentiation was possible whether the substances were of neuronal or endocrine origin. In another study on Burmese pythons, immunohistochemistry was performed and the number of neuronal elements (cell bodies and nerve fibers) was assessed semiquantitatively in the different layers of the small intestine and proximal large intestine, during fasting and 48 h after feeding [1]. Calcitonin-gene related peptide (CGRP), galanin, pituitary adenylate cyclase-activating polypeptide (PACAP), somatostatin, tachykinins and VIP were present in the submucous and myenteric plexus of all examined gut segments (proximal, middle and distal small intestine and proximal large intestine). However, the density was graded on a three-plus (–, +, ++, +++) scale, which makes an objective, reliable comparison between regions and plexuses difficult. With our quantitative assessment, we have found a higher percentage of ChAT-positive than NOS-positive neurons in all regions of the myenteric plexus. The difference was most striking in the oesophagus with 72% ChAT+ and 21% NOS+, and less expressed in the proximal small bowel with 39% ChAT+ and 35% NOS+. On the contrary, in the submucous plexus, in all regions, except for the oesophagus, more NOS+ than ChAT+ neurons were found. For comparison, in the corpus of the guinea pig stomach, 67% of neurons were ChAT and 29% NOS/NADPH-d positive [12]. Further analysis showed that 64% of ChAT+ cells were motoneurons, mostly projecting to the ascending direction, 27% nonmotor, and 9% multitargeted neurons, while 57% of NADPH-reactive neurons were motoneurons, mainly with descending projections, 39% nonmotor, and 4% multitargeted neurons [13]. In our study, we did not analyze projections of enteric neurons, but based on our organ bath experiments, Ach and NO must be released from muscle motoneurons, similarly to the guinea pig. This is confirmed by the presence of ChAT and NOS-positive nerve fibers running in the muscle layer visualized in myenteric plexus preparations.

We could observe VIP-positive neuronal cell bodies in the submucous plexus, but almost none in the myenteric plexus. In the latter, VIP staining was only observed in nerve fibers, with many varicose endings around neurons, probably originating from VIPergic interneurons, and running also in the muscle layer, representing fibers of motoneurons. A similar phenomenon was also described in the myenteric plexus of the guinea pig stomach when axonal transport inhibitors were not applied to increase somatic labeling. [14] The presence of VIP in enteric neurons may be species-specific in the snake, as in an early study in *Vipera aspis*, VIP was identified in nerve terminals and cell bodies in the small intestine, but it could not be shown in *Natrix natrix* and *Natrix maura* [15]. Nitrergic nerves and their colocalization with VIP and galanin were evaluated semiquantitatively in the GI tract of the snake *Thamnophis sirtalis* [8]*.* Nitrergic nerve fibres were most abundant in the myenteric and submucous plexuses and the circular muscle layer of the esophagus and stomach, but rare in the mucosa and longitudinal muscle. The pattern was similar in the small and large intestines, except that here no submucous plexus could be observed. The authors mentioned that only a small proportion of neurons was stained only for NADPH-d (indicating nitrergic neurons), VIP or galanin; in a larger proportion NADPH-d and VIP were colocalized, and the largest amount of neurons was stained for both NADPH-d and galanin. Based on observations in other species and their findings, the authors hypothesized that in this snake, nitrergic neurons are inhibitory motor neurons of the visceral smooth muscle, and are interneurons in the peripheral nervous system of the GI tract. Our results also confirm a frequent colocalization of VIP and NOS in nerve fibers.

Our experiments with the organ bath showed distinct regional motility patterns. The distal stomach presented an intrinsic activity with regular contractions, whereas spontaneous contractions were rarely seen in the proximal stomach and colon. The responses to the electrical stimulation also demonstrated regional differences. While the EFS evoked the largest on-contraction in the distal stomach, the contraction was smaller in the proximal stomach and even less expressed in the colon. This could be readily explained by the distinct functions attributed to the examined GI regions, as the proximal stomach serves more as a reservoir of food, while the distal stomach is responsible for passing the already softened food and digestive enzymes through the pylorus to the small intestine, similarly to guinea pigs or humans [16, 17]. The colon in snakes is probably largely responsible for the storage of feces, as defecation, similarly to feeding, is also not frequent and follows 1–2 weeks after feeding for e.g. in the Burmese python [18]. A study of radiography evaluation of the colon and cloaca of marine and freshwater snakes showed extensive folds in the colon, supporting its main function as a reservoir of feces [19].

There is very little knowledge of the regulation of gastrointestinal motility in snakes. One study used an organ bath setup to examine the effect of various neurotransmitters on the longitudinal muscle layer of the Burmese python stomach and proximal small intestine [1]. In the case of Ach, the basal tonus and/or the amplitude of rhythmic contractions was increased, but the data was not shown, no further detail was given on the activity pattern. In our organ bath experiments, we have confirmed the excitatory effect of externally applied Ach on GI motility of *Crotalus atrox*, as it increased the basal tone in all regions and elevated the frequency and amplitude of spontaneous phasic contractions in the distal stomach. More importantly, by using the muscarinic Ach receptor blocker atropine we have also demonstrated that Ach is an excitatory neurotransmitter increasing muscle activity in all regions. It is released by EFS in all three regions and is largely responsible for the nerve-evoked contractile on-response. Furthermore, it plays a role in the basal tone in the distal stomach, but not in the proximal stomach and the colon, and it contributes to the magnitude of spontaneous contractions in the distal stomach. The observed changes in the tone in the proximal stomach and colon after applying for example L-NAME in our study proves that our experimental setup can readily measure changes in the tone also in these regions. Apparently, the basal secretion of Ach in the case of the proximal stomach and the colon is insignificant, but it is released after stimulation also in these regions. The role of Ach as the main excitatory neurotransmitter in the snake is in accordance with findings in other species for example the fish [20], guinea pig and rabbit [21], and humans [22].

We identified NO as an important inhibitory neurotransmitter in the snake. The NO donor SNP caused a significant relaxation in all regions and suppressed the frequency and amplitude of spontaneous activity in the distal stomach. By means of the NO-synthase inhibitor L-NAME we could also demonstrate that a constant NO release is responsible for a lower basal tone in the proximal stomach and the colon, and for a reduction of the amplitude of the spontaneous contractions in the distal stomach. NO is also released by EFS and reduces the amplitude of the on-response.

Contrary to the study of Holmberg and colleagues which mentions that VIP did not change the motility pattern in the Burmese python [1], we have found that VIP significantly decreased the muscle tone in all three regions, without showing an influence on the spontaneous activity of the distal stomach. This finding is in line with immunohistochemical data of *Thamnophis sirtalis* showing a frequent colocalization of NO and VIP, as both substances seem to be inhibitory neurotransmitters in the snake. VIP is an important inhibitory neurotransmitter of enteric neurons also in fish [10], guinea pigs [23], and humans [24].

Interestingly, the exposition of the tissue specimens to 5-HT revealed region-specific differences. While it increased the tone in the proximal and distal stomach and stimulated the spontaneous activity in the latter, it caused a non-significant relaxation in the colon. 5-HT can act on different 5-HT receptors, and their role is species-specific in mammals. For example, while in rodents the activation of the 5-HT3 receptor plays a role in the peristaltic reflex, this could not be demonstrated in humans [25]. 5-HT has been shown to cause arterial contraction in the rattlesnake *Trimeresurus flavoviridis *via 5-HT1, but not 5-HT2 receptors [26]. However, to the best of our knowledge, the expression and function of 5-HT receptors in the GI tract of snakes have not been explored.

The application of dopamine had no effect on the tone in the proximal stomach and the colon, but it significantly increased the basal tone and frequency and amplitude of spontaneous contractions in the distal stomach. This is a remarkable effect, as in most other species a relaxatory effect of dopamine has been observed, such as in guinea pig [27] and rat jejunum [28], mouse ileum [29], dog [30] or mouse [31] colon. Contrary to mammals, dopamine exerted a stimulatory effect on the contractile activity of beetle hindgut [32]. Nevertheless, the excitatory effect of dopamine in the rattlesnake may be related to the young age of the animals in our study, as our snakes were approx. 9–12 months old, while they normally reach sexual maturity at 3–4 years [33]. A change in the expression of dopaminergic neurons and the effect on small intestinal motility has been described in mice during development [34]. Namely, in 2-day-old pups, dopamine showed a contractile effect in the small bowel, which gradually vanished with age, and switched to a relaxatory response at day 20. Parallel, the D1, D2, and D3 receptor expression increased significantly. Mice lacking the D2 receptor have a significantly accelerated GI transit, suggesting that they have enhanced propulsive reflexes [35]. Although we could demonstrate the presence of dopaminergic neurons in the myenteric plexus by immunohistochemistry, by blocking D2 and D3 receptors with domperidone, we were unable to provoke any change either in the tone or basal motility pattern or in the response to EFS.

In mammals, catecholamines have a complex modulatory role in GI functions, mainly exerting an inhibition on GI motility [36, 37]. Our experiments with isoproterenol, a non-selective ß receptor agonist analogue demonstrated a similar negative effect on motility. It reduced the tone in all regions and suppressed the spontaneous activity of the distal stomach. The use of the ß-receptor antagonist propranolol did not change the basal tone or the spontaneous motility pattern, suggesting a lack of tonic influence on the motility by catecholamines in the snake, at least in isolated tissues. However, a release of catecholamines by EFS may be unmasked by propranolol in the stomach, as after its application the on-response was significantly increased. Our immunohistochemical stainings demonstrated extensive noradrenergic nerve fibers with many varicosities, but no noradrenergic neuronal cells in the stomach and colon. These probably represent sympathetic nerve endings similar to findings in mammals. Interestingly, the staining in the snake for dopamine-ß-hydroxylase, the enzyme converting dopamine to noradrenalin, rarely overlapped with the staining for tyrosine-hydroxylase, although the latter is the enzyme required for the first step of catecholamine synthesis. This is not an unknown phenomenon, as such cells, which acquire dopamine from external sources and convert it further to noradrenalin, were found frequently in circumventricular organs of non-mammalian vertebrates, like lizards [38] and fish [39].

ATP is an important neurotransmitter evoking an inhibitory junction potential accompanied by smooth muscle relaxation in the bowel of some mammalian species such as the guinea pig [40], mouse [41], hamster [42], and human [43], but not in rabbit [44]. In the snake, the P2 purinoreceptor antagonist suramin increased the amplitude of spontaneous contractions in the distal stomach, confirming that although it may not play a role in the basal tone, ATP also could have an inhibitory role in this species.

We have found that fast voltage-gated Na-channels in the snake smooth muscle preparations were insensitive to TTX, seen as an unchanged response to EFS stimulation in the presence of TTX. It is well known from another snake species, the garter snake *Thamnophis sirtalis*, that it has TTX-resistant sodium channels expressed in its skeletal muscle, and the resistance is at least partly based on specific mutations in the tsNaV1.4 [45]. The presence of TTX insensitive channels is thought to be related to co-evolution, as the rough-skinned newt, a salamander that has a high concentration of TTX in its skin, is among the natural preys of this snake [46, 47]. In one early study, tested individuals of this garter snake were shown 2000 times more resistant to the toxin obtained from the skin of the newt compared to the white mouse [46]. Although *Crotalus atrox* is not claimed to be a predator of the rough-skinned newt, six other genera of snakes, including the prairie rattlesnake, *Crotalus viridis*, were described in this study to be 200 times less sensitive to the poison of the newt than white mice [46]. The presence of TTX insensitive Na-channels in the GI tract of the rattlesnake is not completely unique, as such channels have been described in the corpus cavernosum penis from the South American Rattlesnake *Crotalus durissus terrificus,* where the EFS-induced contractions and relaxations were not abolished by TTX [48, 49]. In the same species, the EFS-induced contraction in the skeletal muscle was almost completely blocked by TTX, demonstrating that contrary to *Thamnophis sirtalis*, here the skeletal muscle sodium channels are not resistant to TTX [48]. In the *Crotalus durissus terrificus* corpus cavernosa, the modified Na-free Krebs solution did not have an effect on the EFS-evoked contraction [49]. The authors suggested a release of catecholamines from the endothelium instead of neuronal elements, which may explain the TTX insensitivity of the reaction and the missing effect of the Na-free Krebs solution [49]. Nevertheless, in the case of the EFS-induced relaxation, the release of NO from neuronal elements, which have TTX-insensitive Na-channels, is suspected [48]. In our experiments, we have shown that the on-contraction is mediated by TTX insensitive, lidocaine-sensitive sodium channels, as it was completely abolished by both Na-free Krebs solution and lidocaine. EFS-induced contraction and relaxation were much longer in the snake compared to previous observations in the guinea pig. Similarly, much longer responses to EFS have been observed in the rattlesnake corpus cavernosa compared to marmoset or other mammals [48]. This may be explained by the different current characteristics of TTX-sensitive and insensitive Na-channels. Namely, the activation and inactivation of TTX-insensitive Na-channels are much slower [50]. However, the time characteristics of the responses could be influenced by temperature, which was different in the case of experiments with guinea pig (38 °C) and snake (30 °C) tissue.

In our study, we did not intend to compare the structure and function of the ENS between fasting and fed state but our finding set the basis for such experiments. In the previously mentioned study of Holmberg and colleagues, the total number of neurons did not appear to be different between the fasted/fed state, and no major differences in the innervation pattern and density were seen [1]. Furthermore, the authors stated that there was no difference in the organ bath experiment results when comparing fasted and fed snakes. In our study, the mean duration since the last feeding was 13 days, which means that our snakes can be considered fasting, as it has been shown that the average passage time of a meal of one adult mouse (~ 10% of bodyweight) is 6 days in *Crotalus atrox* [51] and data available from the Burmese python shows that at day 10, the diameter of the small intestine and intestinal nutrient uptake rates return to the fasting state [18]. In accordance with this, only the distal colon contained faecal material in our animals, while the stomach was already empty. The small bowel was completely empty in the majority of snakes; in a few cases, some short bone remnants, but no larger amounts of digesta were visible.

## Conclusion

The detailed anatomical and functional analysis of the ENS of the rattlesnake *Crotalus atrox* provides new insights into the similarities and differences between species. While many neurotransmitters have identical functions to other species, some show distinct effects. Further studies on receptor expression in the GI tract of snakes are necessary to provide explanation for the observed peculiarities compared to other species.

## Methods

### Animals

Juvenile western diamondback rattlesnakes of either sex (*Crotalus atrox,* n = 35), between 9–12 months old, were used for the experiments. Snakes with a body weight of 39 [32/50] g and body length of 39 [34/42] cm (excluding the rattle) were obtained from the in-house animal breeding facility at the Chair of Zoology of the Technical University of Munich. Animals were kept at a temperature of 22–30 °C with a 12 h/12 h light/dark cycle. Water was provided ad libitum. The last feeding with a pre-killed mouse was 13.1 ± 0.8 days before the experiments. Care and maintenance of the animals followed the established guidelines for venomous snakes [52]. All animal work was conducted according to the German guidelines for animal care and welfare (Deutsches Tierschutzgesetz) and approved by the Bavarian state ethics committee (Regierung Oberbayern, which serves as the Institutional Care and Use Committee for the Technische Universität München) according to §4, §7 and §11 Deutsches Tierschutzgesetz under reference number 55.2–1-54–2532.6–9-12. Animals were initially anesthetized with isoflurane in an induction chamber. After the tail-pinch reflex ceased, snakes were secured with u-shaped pins to the Sylgard (Dow Corning, Wiesbaden, Germany) floor of a large Petri dish. Intramuscular injection of a combination of Ketamine hydrochloride (40 mg/kg; Ketamine 100 mg/ml, Ketavet, Zoetis Deutschland GmbH, Berlin, Germany) and Xylazine hydrochloride (20 mg/kg; Rompun 2%, Bayer Vital GmbH, Leverkusen, Germany) ensured deep anesthesia. Thereafter, the animals were decapitated and the body was opened up to the cloaca. The gastrointestinal tract, including oesophagus, stomach, small and large intestine, liver, and gall bladder were removed and placed in ice-cold Krebs solution.

### Preparation of whole-mount tissue

**Fig. 6:**
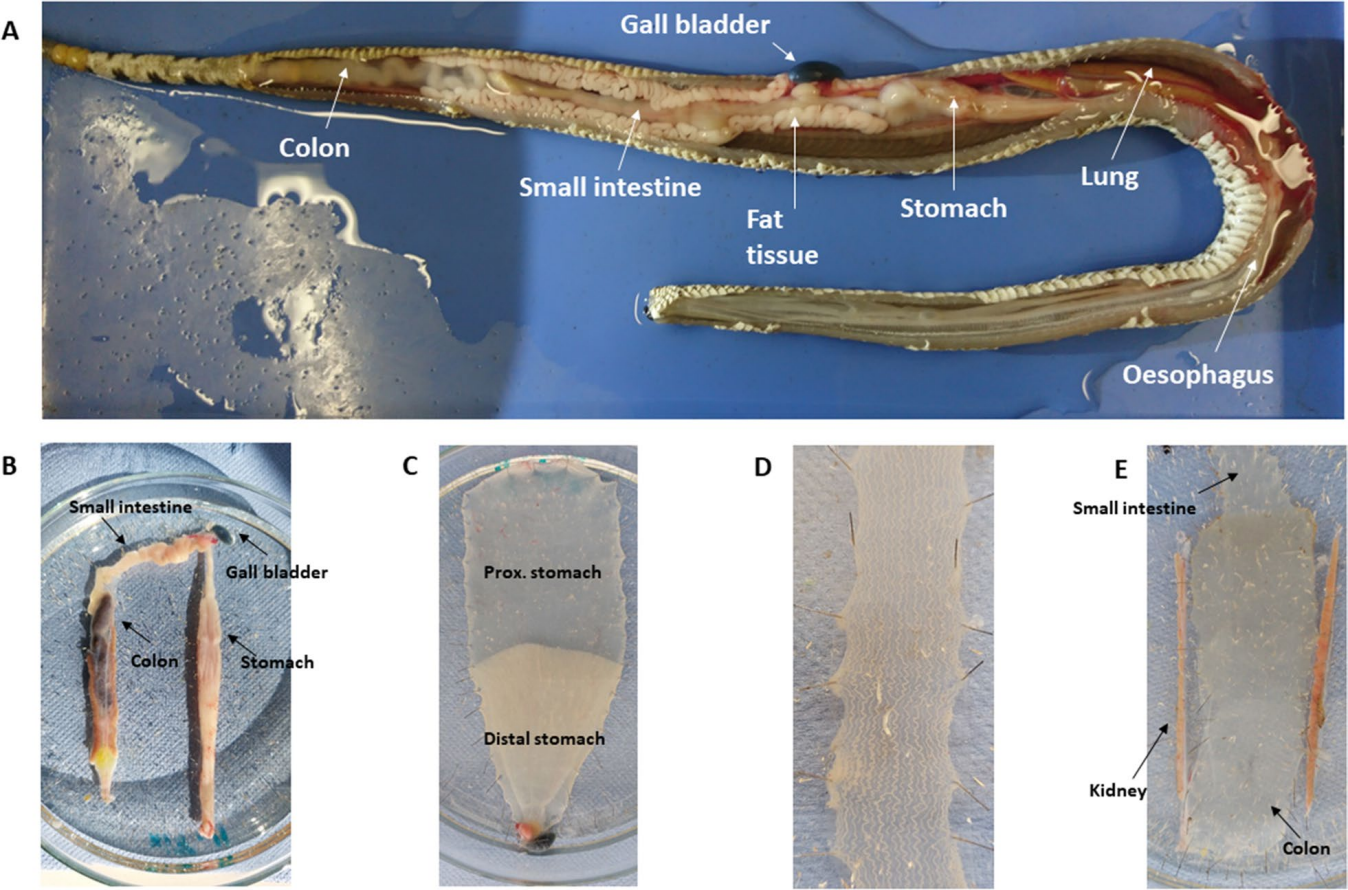
Anatomy of *Crotalus atrox*. **A** Internal organs in their original position. The whole GI tract is empty in this animal. **B** Gastrointestinal tract removed from another snake. Note the empty stomach and small intestine. In the colon faecal material is visible in this case. **C** Stretched stomach, with a clear difference visible between the proximal and distal regions. **D** Stretched small intestine. **E** Stretched colon

The different regions of the gastrointestinal tract were separated, pinned, and stretched in Petri-dishes filled with 4 °C Krebs solution (Fig. 6). Seven gut regions have been selected for analysis: oesophagus (the segment proximal to the heart), proximal and distal stomach (separated by a visible difference in the texture of the mucosa and underlying muscle layer), proximal small intestine (immediately after the pylorus), distal small intestine (proximal to the kidneys), proximal colon (starting from the proximal end of the kidneys) and distal colon (defined by a different texture of the mucosa) (Fig. 6). At first, the total length (I total) of each region were measured. The specimens were then opened at the mesenteric border. To determine the stretch factor, a piece with approx. 1 cm length was cut from each part of the GI tract and the length (l unstretched) and width (d unstretched, indicating the circumference) were determined before stretching the tissue. The tissue pieces were then pinned with maximal stretch and their length (l stretched) and width (d stretched) was measured. The stretch factor was calculated for each region by using the formula: I stretched × d stretched ∕ I unstretched × d unstretched. The stretched tissues were fixed for 4 h at room temperature with 3% phosphate-buffered formaldehyde solution followed by 3 × 10 min rinsing with phosphate buffer solution. From three animals, a piece measuring 1.5 × 2 cm was cut out from each of these regions and the submucous plexus and the myenteric plexus were prepared under stereomicroscope.

### Transverse sections

For the transverse sections, 1 cm long sections of the seven regions of the intact, unopened gut were cut out, fixed for 4 h the same way as the whole mount preparations, followed by 3 × 10 min rinsing with phosphate buffer solution and incubated overnight in sucrose-solution (30% sucrose in PBS/NaN3). The tissue pieces were shock-frozen with dry ice, embedded in Shandon Cryomatrix™ (Thermo Fisher Scientific, Darmstadt, Germany) and 15 µm frozen sections were prepared with a cryostat (Leitz 1720). The sections were mounted on slides (SuperFrost® Plus; Thermo Fisher Scientific, Darmstadt, Germany).

### Immunohistochemistry

Whole-mount tissues were first incubated at room temperature for 1 h in Triton X-100 (PBS/NaN_3_/4% horse serum/0.5% Triton X) followed by 16 h and 2 h incubation with the primary and secondary antibodies, respectively. Primary antibodies were human anti-HU (1:10,000; AI01cPR), rabbit anti-NOS (1:20,000; Enzo Life Sciences; 210 501 R025), goat anti-choline-acetyltransferase (ChAT 1:500; Merck-Millipore AB 144 P); mouse anti-VIP (1:20,000; east acres; MaVIP), sheep anti-tyrosine-hydroxylase (TH, 1:5000; Chemicon, AB1542) and rabbit dopamine-ß-hydroxylase (DHB, 1:1000; Affiniti, DZ 1020). Secondary antibodies were donkey anti-human IgG DyLight488 (1:200; Dianova; 709–485-194) donkey anti-human IgG AMCA (1:50; Dianova; 709–155-149) donkey anti-rabbit Cy2 (1:200; Dianova; 711–225-152); donkey anti-rabbit Cy3 (1:500; Dianova; 711–165-152); donkey anti-goat Cy3 (1:500; Dianova; 705–165-147), donkey anti-mouse Cy5 (1:500; Dianova; 715–175-151), donkey anti sheep Cy2 (1:200; Dianova, 713–225-147. Finally, specimens were rinsed in phosphate buffer saline, mounted on slides (SuperFrost® Plus; Thermo Fisher Scientific), cover slipped with the mounting medium (antifade solution, containing 80% Glycerol 20% PBS/NaN3 (0.1%), pH = 7.00) and left at least 4 h at 4 °C before examination under the fluorescent microscope.

Transverse sections were incubated also for 16 h and 2 h with the primary and secondary antibodies. However, here the primary antibodies were the human anti-HU (1:10,000; AI01cPR) and the rabbit anti-Human PGP9.5 (1:30,000; Accurate Chemical; UCRA95101), the secondary antibodies donkey anti-human IgG DyLight488 (1:200; Dianova; 709–485-194) and donkey anti-rabbit IgG Cy3 (1:500; Dianova; 711 165 152).

### Antibody characterization

The specificity of the human-anti-Hu antibody [53], rabbit anti-NOS and rabbit dopamine-ß-hydroxylase [12], goat anti-choline-acetyltransferase [54], mouse anti-VIP [55] was previously demonstrated by our group. Sheep anti-tyrosine-hydroxylase was previously published by our group here [56]. The latter was tested parallel to mouse anti-tyrosine-hydroxylase (Merck, T 1299) in our lab to confirm that they stain the same structures.

### Data analysis

Four regions (one region from each quarter of the tissue) were selected and analyzed at 20 × magnification. Images were taken from all selected regions for all four stainings (HU, VIP, CHAT, NOS) with the corresponding wavelengths, and finally, all images were overlayed in CellP (Olympus Soft Imaging Solutions, Hamburg). As in the VIP staining of the myenteric plexus no neuronal bodies were visualized, here only the HU, ChAT, and NOS stainings were overlayed. Neuronal cell bodies were manually counted with the software CellP. By means of the stretch factor (stretch factor = [length × width of stretched tissue]/[original length and width of tissue]) and the surface of the examined area (887.5 µm × 665.6 µm) the density of neurons per cm^2^ was calculated. (Neuronal density [1/cm^2^] = number of neurons/examined surface [= 0.0059072 cm^2^]  × stretch factor) The total number of neurons for each region was calculated by multiplying the neuronal density of that region with the total surface of the region (I total × d unstretched).

### Statistical analysis

The statistical analysis was performed with the software R and Sigma Plot 12.5. After the normality test (Shapiro–Wilk test) and the test for homogeneity of variances (Fligner-test), one-way ANOVA was used, followed by a Tukey post-hoc test. In the case of non-normal distribution Kruskal–Wallis test was followed by Dunn`s test with Bonferroni correction.

### Organ bath experiments

#### Preparation

The entire gastrointestinal tract (Fig. 6) was removed and immediately immersed in ice-cold, carbogen-bubbled (95% O2, 5% CO2) Krebs solution (pH 7.4, composition in mmol L-1: 117 NaCl, 4.7 KCl, 2.5 CaCl2 (2H2O), 1.5 MgCl2 (6H2O), 25 NaHCO3, 1.2 NaH2PO4, and 11.0 Glucose). The stomach was cut up longitudinally, rinsed in Krebs solution, and fixed mucosal side up with metal pins in Sylgard-coated Petri dishes. With the mucosa still on, muscle strips (0.5 cm wide and 1 cm long in the stomach and 1 cm^2^ in the colon) were cut along the circular muscle axis of the proximal and distal stomach and the colon under an Olympus SZ51 stereomicroscope (Olympus, Hamburg, Germany), and mounted in a four-chamber, 25 mL automatic organ bath (Panlab, Barcelona, Spain), The method was similar to our previous studies with guinea pig tissue with a few adaptations to the snake [57, 58].

The strips were maintained constantly in carbogen-bubbled Krebs solution at 30 °C and pH between 7.3–7.4. The muscle strips were attached to an isometric tension transducer connected to a Quad Bridge and a PowerLab 4/35 analog/digital converter (ADInstruments, Oxford, UK). Contractile activity was recorded and analyzed employing LabChart 7 software (ADInstruments) on a computer. After setting a preload of 20 mN, tissue preparations were equilibrated for 60 min. Electrical field stimulation (EFS) was performed with a Grass SD9 stimulator (100 V, 10 Hz, pulse width of 0.5 ms, 10 s). Viable tissues typically responded with a biphasic response, with an initial contraction starting during the 10 s of stimulation called contractile on-response, followed by a relaxation, starting a few seconds after the end of the stimulus, called late inhibition. In other cases, the late response was completely missing, or it was also a contraction. Tissues were thoroughly rinsed after each electrical stimulation.

#### Drug applications

The list of the applied drugs is shown in Table 5.

**Table 5:** Information on chemicals used in the organ bath experiments

Drug	Concentration in organ bath	Company and product nr
Acetylcholine (ACh)	10 µM	Sigma-Aldrich, A-6625
Atropine (ATR)	1 µM	Sigma Aldrich, A-4529
Dopamine	10 µM	Sigma-Aldrich, H-4381
Isoproterenol	10 µM	Sigma-Aldrich, I-5627
Lidocaine	1 mM	Sigma-Aldrich, L-5647
N(γ)-Nitro-L-Arginin Methyl Ester (L-NAME)	1 mM	Sigma/Merck, N-5751
Sodium Nitroprusside (SNP)	100 µM	Sigma-Aldrich, S-0501
Propranolol	5 µM	Calbiochem, 537075
Serotonin (5-HT)	1 µM	Sigma-Aldrich, H-7752
Suramin	100 µM	Sigma-Aldrich, S-2671
Tetrodotoxin	0.5 µM	Biotrend, BN0518
Vasoactive Intestinal Peptide (VIP)	0.1 µM	Peninsula Lab., 7150

#### Data analysis

The changes in muscle tension after EFS or evoked by different drugs were compared to the baseline tension before EFS or before adding the drugs and expressed as ∆mN. In the case of distal stomach muscle strips the effect of the drug on the amplitude and frequency of spontaneous phasic contractions was also analyzed. For paired experiments, paired Student t-test or a signed rank test in case of data with a non-Gaussian distribution was used. For multiple comparisons, One Way Analysis of Variance, or in the case of data with non-Gaussian distribution, Kruskal–Wallis One Way Analysis of Variance on Ranks was applied. Statistical significance was determined as *p* < 0.05. Values are expressed as mean $\pm$ SEM in case of frequency, and as median [25%/75%] in case of all other values (for better comparability we present the data in median also in case of a normal distribution).

### Neuroimaging experiments

#### Tissue preparation

The proximal colon of *Crotalus atrox* was removed, cut along the mesenteric border, and washed several times with cold Krebs solution. The tissue was stretched on a dissecting dish lined with Sylgard 184 (Dow Corning, Wiesbaden, Germany) containing ice-cold oxygenated Krebs solution that was exchanged every 10 min. In order to obtain a myenteric plexus preparation, the tissue was stretched serosal side up using insect pins so that the serosa, a web-like structure, could be removed. Subsequently, the tissue was flipped and stretched again allowing the mucosa and submucous plexus to be gently removed, thus leaving the myenteric plexus situated between the circular and longitudinal muscle layers. The final preparation (10 × 20 mm) was pinned on a silicone ring that was placed in a recording chamber with a 42 mm diameter glass bottom revealing the serosal side of the tissue facing upward.

#### Imaging technique (MSORT)

To confirm the results from the organ bath, the myenteric plexus was studied using Multi-Site Optical Recording Technique (MSORT), described in detail in previous publications from our group [59, 60]. For this technique, the chamber was mounted onto an epifluorescence Olympus IX 50 microscope (Olympus, Hamburg, Germany) equipped with a green LED light source (LE T S2W, Osram, Munich, Germany)). The recording chamber was constantly perfused with 30 °C Krebs solution and gassed with Carbogen (5% CO_2_, 95% O_2_) at a pH of 7.4. Using local pressure application (1.5 bar, 999 ms) through a microejection pipette (Science Products, Hofheim, Germany) attached to a micromanipulator, ganglia were stained with 20 µM Di-8-ANEPPS (1-(3-sulfonatopropyl)-4-[beta[2-(di-*n*-octylamino)-6-naphthyl]vinyl]pyridinium betaine), a fluorescent voltage-sensitive dye (Biozol, Eching, Germany) retained in the outer leaflet of cell plasma membranes allowing for visualization individual cells. Neurons stained with Di-8-ANEPPS were visualized using an × 40 oil immersion objective (UAPO/340, NA = 1.4; Olympus), 520-550 nm excitation interference filter, a 565 nm dichroic mirror and 580 nm barrier filter (Olympus). Changes in membrane potential are evident as they are linearly related to changes in fluorescent intensity (ΔF/F) [61]. Using the Neuroplex-CCD system program, acquisition and processing of neuronal activity were obtained with a resolution of 80 × 80 pixels at a frequency of 1 kHz (RedShirt Imaging, Decatur, GA, USA).

In order to test the viability of neurons in a ganglion, electric stimulation on an interganglionic fiber tract was carried out using a Teflon-coated platinum electrode (25 µm diameter, 101R-1 T, Science Products) connected to a stimulator (S88, Grass-Telefactor). Single electric pulses had a duration of 600 µs, 30µA current, and a recording duration of 500 ms. A ganglion was deemed viable following EFS in the presence of an action potential (refer to [9] for detailed examples).

Upon demonstrating neuron viability, it was necessary to confirm the TTX-insensitivity of the EFS. The tissue was perfused with 100 mL of 1 µM Tetrodotoxin (Bio-techne, Wiesbaden, Germany) gassed with Carbogen (5% CO_2_, 95% O_2_) for 15 min directly followed by electric stimulation. In the next step the tissue was perfused with 100 mL of 1 mM lidocaine (Sigma-Aldrich) gassed with Carbogen (5% CO_2_, 95% O_2_) for 15 min followed by EFS before and after a 1 h washout period. All traces were exported for analysis using Igor Pro 8 (Wavemetrics, Inc., Lake Oswego, OR, USA).

## Data Availability

The datasets used and analyzed during the current study are available from the corresponding author on reasonable request.
